# Cerebral astrocytoma in a sheep

**Published:** 2017-09-15

**Authors:** Ghasem Farjanikish, Azizollah Khodakaram-Tafti, Omid Dezfoulian

**Affiliations:** 1 Department of Pathobiology, Faculty of Veterinary Medicine, Lorestan University, Khorramabad, Iran;; 2 Department of Pathology, School of Veterinary Medicine, Shiraz University, Shiraz, Iran

**Keywords:** Astrocytoma, Histopathology, Immunohistochemistry, Sheep

## Abstract

Astrocytoma as one of the most common central nervous system (CNS) tumors is rarely reported in veterinary literature. A 7-year-old Persian Lori-Bakhtiari ewe was presented to the clinic with a two months history of progressive blindness, nystagmus to the right, bilaterally decreased pupillary reflexes, head pressing and paddling. At necropsy, a whitish well-circumscribed mass with dimensions of 3.50×2.50×1.50 cm was observed in the dorsal parietal lobe of the left cerebral hemisphere. Microscopically, the mass was well-circumscribed and highly cellular, consisted of round to elongated cells with scant and vacuolated cytoplasm with few, flaccid processes. The nuclei were round to oval with densely stippled chromatin and indistinct nucleoli. Immunohistochemical analyses showed positive staining for vimentin, S100 and glial fibrillary acidic protein. Definitive diagnosis of cerebral protoplasmic astrocytoma was made on the basis of the histopathological and immunohistochemical findings. This type of neoplasm should be included in the differential diagnosis of CNS lesions in the sheep.

## Introduction

Gliomas are the most common type of neuroectodermal-origin tumors in humans and animals.^1^^,^^2^ Glioma is a term including ependymoma, oligodendroglioma, glioblastoma, astrocytoma and their various subtypes and combinations.^3^ Astrocytoma is one of the most common central nervous system (CNS) tumors in animals. The tumor, being well-differentiated, is a diffusely infiltrative neoplasm of astrocytes and occurs most commonly in the pyriform lobe, convexity of the cerebral hemisphere, thalamus and hypothalamus, midbrain and rarely in the cerebellum and spinal cord.^1^^,^^4^^,^^5^ Astrocytomas in humans are heterogeneous groups of malignant neoplasms with clinical, radiographical and histological features with typically having a bimodal age distribution. Brainstem is more commonly affected in childhood, whereas cerebral astrocytomas are more common in middle age.^6^

Neoplastic cells typically have obvious astrocytic appearances histologically, although this can sometimes be difficult to distinguish from reactive astrocytosis, particularly in biopsy specimens taken from the periphery of an infiltrating mass. All astrocytic tumors share the common characteristic of being highly cellular with peripheral invasion into the neuroparenchyma and stain consistently immunopositive for glial fibrillary acidic protein (GFAP).^6^

Astrocytoma has been reported so far in dog (especially brachycephalic breeds), cat, pig, cattle and goat.^6^^-^^10^ The present study describes the clinical, histo-pathological and immunohistochemical characteristics of an astrocytoma in the cerebrum of a sheep.

## Case Description

A 7-year-old Persian Lori-Bakhtiari ewe was presented with a 2-month history of blindness and behavioral disorders. Neurological examinations revealed nystagmus to the right, bilaterally decreased pupillary reflexes, head pressing and paddling.

**Fig. 1:**
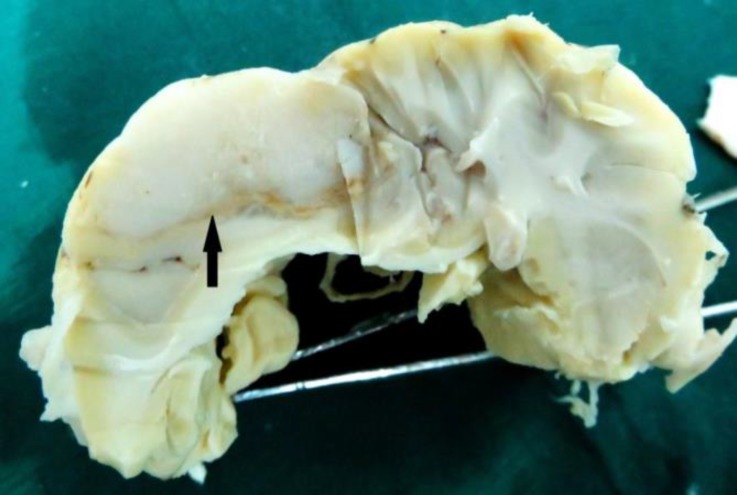
The mass (arrow) was firm and well-circumscribed with a soft and homogeneous appearance suspected to astrocytoma

The ewe was euthanized because of poor prognosis and a post-mortem examination was performed. At necropsy, gross pathological findings were limited to the brain. A whitish, firm and well-circumscribed mass, with a regular surface of approximately 3.50×2.50×1.50 cm was observed in the dorsal parietal lobe of the left hemisphere. The cut surfaces were soft, gray and homogeneous (Fig. 1). No macroscopical involvement of the meninges or periosteum of the skull and no macroscopically observed metastases were found in other organs.

The appropriate samples of the tumoral mass were fixed in 10% neutral buffered formalin, dehydrated in graded ethanol, cleared in xylene and embedded in paraffin wax. The sections with 5 µm thickness were stained with hematoxylin and eosin (H&E) and studied microscopically. In order to establish a final diagnosis of the neoplasm, the following antibodies were applied in appropriate dilutions on the brain sections: anti-protein S100 (polyclonal rabbit code No.IS504; Dako, Glostrup, Denmark), anti-glial fibrillary acidic protein (GFAP, Clone CF2, 1/300; Dako), p53 (Clone DO-7; Dako), anti-epidermal growth factor receptor (EGFR, Clone H11, 1/100; Dako), epithelial membrane antigen (EMA, Clone E29, RTU, Dako), vimentin (Clone V9; Dako), neuron specific enolase (NSE, Clone BBS/NC/VI-H14, RTU, Dako) and oligodendrocyte transcription factor (Polyclonal antibody anti-olig 2, EMD Millipore Corp., Billerica, MA). Primary antibody was detected by EnVision™ + /HRP Mouse code K4001 (Dako) and visualized with 3,3′-diaminobenzidine (DAB) as chromogen. Counterstaining was performed with Mayers hematoxylin.

**Fig. 2:**
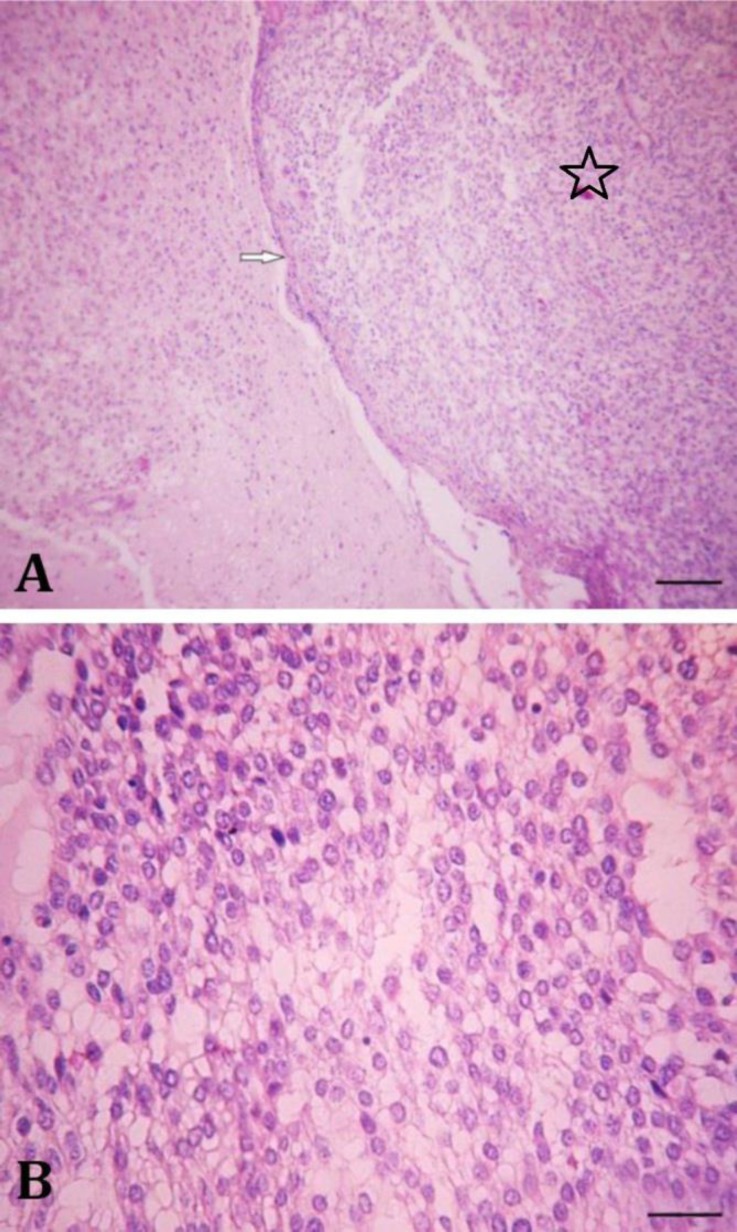
**) **Microscopical examination revealed a densely cellular and encapsulated mass (arrow). Cellular density within these areas was higher than that of normal areas of nervous tissue (Asterisk), (H & E, Bar = 150 µm); B) The neoplastic cells have scant and vacuolated cytoplasm with few, flaccid processes and round to oval nuclei (H & E, Bar = 40 µm

Microscopical lesions were limited to the cerebrum. The tumor was well-circumscribed, densely cellular and encapsulated. Cellular density within these areas was higher than that within normal areas of nervous tissue. The tumor was composed of neoplastic cells with scant and vacuolated cytoplasm with few, flaccid processes and round to oval nuclei with densely stippled chromatin and indistinct nucleoli (Fig. 2).

**Fig. 3:**
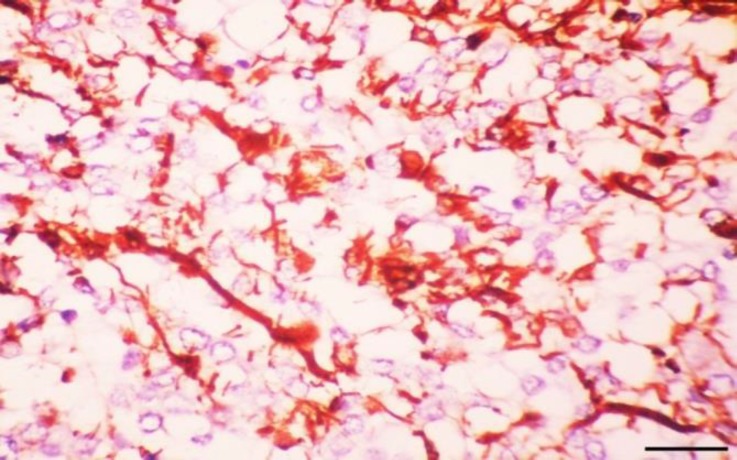
The neoplastic cells stain moderately with glial fibrillary acidic protein (Bar = 20 µm).

**Fig. 4:**
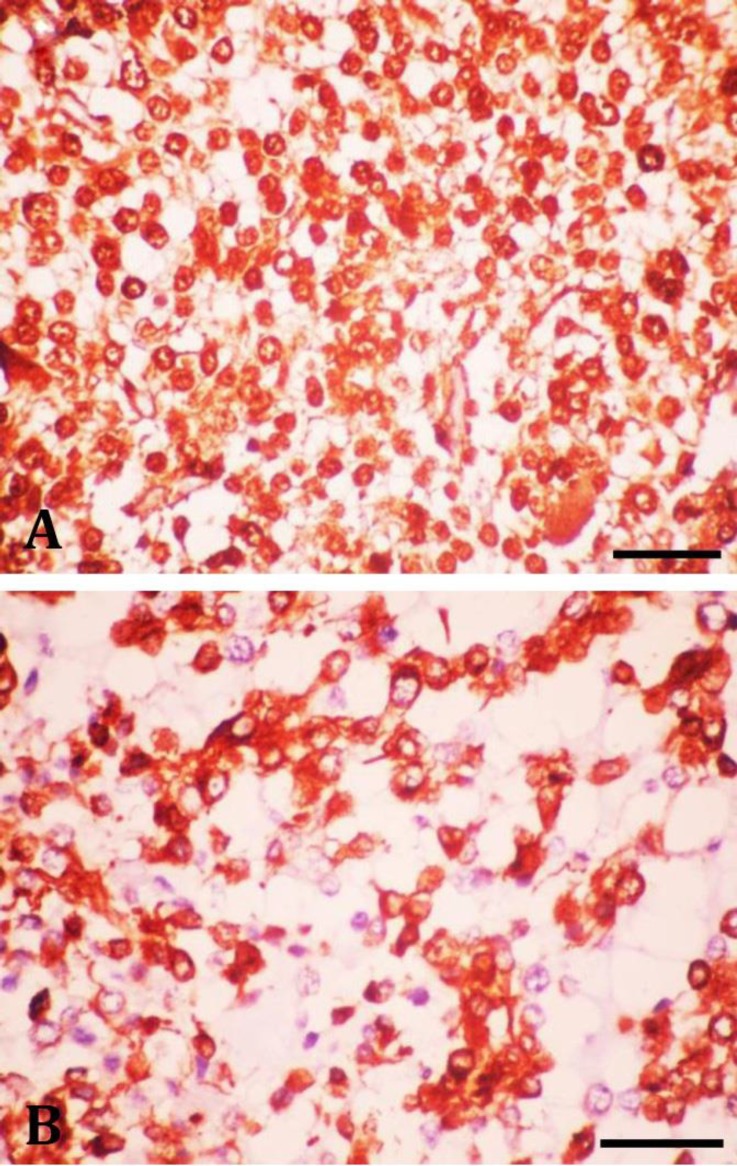
A) The neoplastic cells show strong positive reactivity for S100 (Bar = 25 µm); B) The neoplastic cells express vimentin (Bar = 20 µm).

The central portion of the neoplasm contained multiple necrotic foci and vascular proliferation. The remaining neoplastic cells possessed intermediate cellular morphologies and rare mitotic figures were observed. No invasion was detected in adjacent brain tissue. Immunohistochemically, the neoplastic cells were labelled moderately for GFAP (Fig. 3) and strong diffusely for S100 and vimentin (Fig. 4), but had no expression to other markers.

Based on histopathological and immunohistochemical findings of the mass, the tumor was diagnosed as a protoplasmic form of astrocytoma.

## Discussion

Primary tumors of the CNS are rarely reported in domestic animals, except in the dog.^4^^,^^7^^,^^11^^,^^12^ Glial tumors such as astrocytoma are the most common primary CNS tumors in humans, dogs and cats.^1^^,^^2^ To date, very few cases of primary CNS tumors have been reported in sheep including medulloblastoma in lambs,^13^ ependymoma in a Suffolk sheep aged <1 year,^14^oligodendroglioma in a 1-year-old male Iranian fat-tailed sheep^15^and more recently, glioblastoma with oligodendroglioma component in a 6-year-old Sardinian breed ewe.^16^

The world health organization classification is widely adapted to classify astrocytomas, in which the groups are designated as low-grade gliomas (grade I or II), diffuse astrocytoma (grade II), anaplastic astrocytoma (grade III) or glioblastoma multiforme (grade IV). The tumor grade of astrocytoma includes fibrillary, gemistocytic and protoplasmic types.^1^^,^^5^^,^^19^

Protoplasmic astrocytomas are unusual and rare variants of diffuse astrocytomas composed of stellate-shaped astrocytes with short and delicate processes and a prominent background of microcysts.^12^ Histological grading criteria include cell density, cellular and nuclear pleomorphism, nuclear hyperchromatism, presence of mitotic figures and necrosis. According to these criteria, the present case falls into the diffuse protoplasmic type. In the present case, the tumor seems to be predominantly composed of neoplastic cells showing scant and vacuolated cytoplasm with few, flaccid processes, resembling at least a protoplasmic astrocytoma. This subtype is considered a rare variant of diffuse astrocytoma.

In humans, astrocytoma is usually single tumor most often occurs in the third and fourth decades of life and males are affected more often than females. Protoplasmic astrocytoma is unusual and is most often found in the cerebrum in children and young adults.^19^ In this ewe, the tumor was located in the dorsal parietal lobe of the left hemisphere.

Clinical signs in animals with astrocytoma vary depending on the location of the tumor in the CNS. The most common signs include behavioral changes, ataxia, tetraparesis, seizures, circling and abnormal cranial nerve and proprioceptive reflexes.^17^ The animal in the present report showed neurological signs including nystagmus to the right, bilaterally decreased pupillary reflexes, head tilt, head pressing and paddling.

In humans, genetic alterations such as inactivation of p53, amplification and rearrangement of platelet-derived growth factor receptor A and changes in integrin expression have been associated with the progression of astrocytic tumors from low to high grade. Radiation can also induce tumors in the CNS, usually after years of radiation therapy, including poorly differentiated sarcomas, gliomas and meningiomas.^18^^,^^19^ Some reports have demonstrated that occurrence of astrocytoma in dogs and cats can be related to genetic alterations such as inactivation of p53 and overexpression of EGFR.^20^^,^^21^

The treatment of choice for brain tumors including surgical removal^22^and computerized tomography has been used to diagnose and localize tumors and facilitate their removal.^23^ The treatment of astrocytoma is a challenge in veterinary medicine. Corticosteroids are frequently used as palliative therapy and can reduce the vascular permeability, exert cytotoxic effects on tumors, inhibit tumor formation and decrease cerebrospinal fluid production.^24^ There is a controversy concerning the benefits of surgical excision of CNS tumors in animals. However, whether in humans or animals, surgical debulking followed by radiation therapy provides the best prognosis for most tumors.^23^

In this case, histopathological and immunohisto-chemical findings confirm astrocytoma accordance with the well-documented diagnosis for protoplasmic astrocytoma in the literature.^5^^,^^10^^,^^11^^,^^21^^,^^28^ Most astrocytomas are GFAP, S100 and vimentin positive. Interestingly, cytokeratin and epidermal growth factor are sometimes positive as well. Differential diagnoses for these low-grade tumors include oligodendroglioma, higher-grade tumors, fibrillar ependymoma, reactive gliosis, demyelinating disease and cerebral infarcts. It is realized that expression of EMA is demonstrated in some glial tumors in animals.^25^ However, this marker is considered as a potentiated antibody for differentiation of ependymoma from glial tumors in humans.^26^ Moreover, EGFR overexpression appears in nearly half of individuals with glioblastoma multiforme.^27^ Herein, our immunohistochemical data in sheep reported in this work are largely in agreement with those reported in other domestic animals and also, in humans.^6^^,^^23^^,^^28^

On the basis of histological and immuno-histochemical findings, a diagnosis of protoplasmic astrocytoma was made which is a rare variant of diffuse astrocytoma. To the best of our knowledge, this is a rare reported case of astrocytoma in sheep. This type of neoplasm should be included in the differential diagnosis of CNS lesions in the sheep.

## References

[1] Koestner A, Higgins RJ (2008). Tumors in domestic animals.

[2] Santana FF, Serakides R, Graca DL (2002). Pilocytic astrocytoma in a cat. Vet Pathol.

[3] McKeever PE, Boyer PJ, Mills SE (2004). The brain, spinal cord, and meninges. Steenbergs diagnostic surgical pathology.

[4] Summers BA, Cummings JF, Lahunta A (1995). Veterinary neuropathology.

[5] Wilson PE, Oleszek JL, Clayton GH (2007). Pediatric spinal cord tumors and masses. J Spinal Cord Med.

[6] Burger PC, Scheithauer BW (1994). Tumors of the central nervous system.

[7] Fankhauser R, Luginbuhl H, McGrath JT (1974). Tumors of the nervous system. Bull World Health Organ.

[8] Higgins RJ, Theilenand GH, Madewell BR (1987). Tumors of the nervous system Part I Pathology. Veterinary cancer medicine,.

[9] Cordy DR, Moulton JE (1990). Tumors of the nervous system and eye. Tumors in domestic animals.

[10] Delas Mulas JM, Bautista MJ, Fernando CM (1996). Fibrillary astrocytoma in a goat: Pathologic, immunohistochemical, and ultrastructural study. J Vet Diagn Invest.

[11] Frenier SL, Kraft SL, Moore MP (1990). Canine intracranial astrocytomas and comparison with the human counterpart. Compend Contin Educ Small Anim.

[12] Meuten DJ (2008). Tumors in domestic animals.

[13] Cotchin E (1975). Spontaneous tumors in young animals. Proc R Soc Med.

[14] Elsinghorst TA (2003). First cases of animal diseases published since 2000. Sheep. Vet Q.

[15] Derakhshanfar A, Mozaffari AA (2010). First report of oligodendroglioma in a sheep. J S Afr Vet Assoc.

[16] Pintus D, Marruchella G, Masia M (2016). Glioblastoma with oligodendroglioma component in a ewe. J Vet Diagn Invest.

[17] Zaki FA, Hurvitz AI (1976). Spontaneous neoplasms of the central nervous system of the cat. J Small Anim Pract.

[18] Kim KE, Kim KU, Kim DC (2009). Cytogenetic characterizations of central nervous system tumors: The first comprehensive report from a single institution in Korea. J Korean Med Sci.

[19] Lopes MS, Vandenberg SR, Fletcher DM (2002). Tumors of the central nervous system. Diagnostic histopathology of tumors.

[20] Aloisio F, Jonathan ML, Edwards JF (2008). Immuno-histochemical features of a feline spinal cord gemistocytic astrocytoma. J Vet Diagn Invest.

[21] Stoica G, Kim HT, Hall DG (2004). Morphology, immunohistochemistry, and genetic alterations in dog astrocytomas. Vet Pathol.

[22] De Lahunta A (1977). Veterinary neuroanatomy and clinical neurology.

[23] Siso S, Lorenzo V, Ferrer I (2003). An anaplastic astrocytoma (optic chiasmatic-hypothalamic glioma) in a dog. Vet Pathol.

[24] Montgomery DL (1994). Astrocytes: Form, functions, and roles in disease. Vet Pathol.

[25] Yamada M, Nakagawa M, Yamamoto M (1998). Histopathological and immunohistochemical studies of intracranial nervous-system tumors in four cattle. J Comp Pathol.

[26] Hitchcock E, Morris CS (1987). Cross reactivity of anti-epithelial membrane antigen monoclonal for reactive and neoplastic glial cells. J Neurooncol.

[27] Voelzke WR, Petty Glenn WJ, Lesser GJ (2008). Targeting the epidermal growth factor receptor in high-grade astrocytomas. Curr Treat Options Oncol.

[28] Da Silva EO, Dos Reis AF, Bracarense1 AP (2010). Canine cerebellar protoplasmic astrocytoma: clinical, histo-pathological and immunohistochemical features. Braz J Vet Pathol.

